# Trends of infectious diseases, epidemic patterns, and the association with meteorological events: 2500 years of evidence from an observational study

**DOI:** 10.7189/jogh.15.04254

**Published:** 2025-09-05

**Authors:** Qiao Liu, Chenyuan Qin, Shimo Zhang, Jue Liu

**Affiliations:** 1 Department of Epidemiology and Biostatistics, School of Public Health, Peking University, Beijing, China; 2 Health Emergency Management Center, Peking University, Beijing, China; 3 Institute of Environmental Medicine, Peking University, Beijing, China; 4 Key Laboratory of Epidemiology of Major Diseases (Peking University), Ministry of Education, Beijing, China

## Abstract

**Background:**

Meteorological factors are known to influence the transmission of infectious diseases. Studying historical epidemics in ancient China provides valuable insights into how environmental stressors shaped public health, with implications for modern disease control. We aimed to quantitatively assess the relationship between meteorological events and epidemic severity in China from 674 BC to 1911 AD.

**Methods:**

We extracted data from ‘A Compendium of Chinese Meteorological Records of the Last 3000 Years’. We digitised epidemic events, consequences, and associated meteorological conditions and categorised them into ordinal levels. We used descriptive statistics and multivariable logistic regression to analyse annual patterns and associations across historical periods.

**Results:**

We identified 5338 epidemic-related records. While the number of reported epidemics increased over time, the proportion associated with meteorological events declined by 0.24% (95% confidence interval (CI) = 0.17–0.31) per decade between 1451 and 1911. Flood frequency was associated with higher epidemic severity: each additional flood increased the odds of moderate (*vs.* mild) consequences by 42% (95% CI = 8–87) and severe (*vs.* moderate) consequences by 46% (95% CI = 12–91). Droughts similarly raised the risk of severe consequences by 23% (95% CI = 7–41). Famines were also linked with heightened epidemic severity and were among the most commonly co-reported meteorological events, along with drought.

**Conclusions:**

As global climate intensifies, the historical relationship between environmental stressors and epidemic severity offers crucial lessons for modern public health. Regions vulnerable to climate extremes may require targeted, climate-informed epidemic preparedness and response strategies.

Meteorological factors have been evidenced to play a crucial role in the transmission of various infectious diseases. A multi-city study in China has revealed the potential impact of weather conditions on the spread of COVID-19 [1], while seasonal diseases such as hand, foot, and mouth disease are also strongly influenced by meteorological conditions [2]. Temperature and precipitation have been shown to affect malaria incidence across 57 countries and territories [3]. Natural disasters such as floods also significantly impact disease transmission patterns. A study involving 168 countries revealed a strong association between flood disasters and the upsurge of different infectious diseases [4]. Such disasters can catalyse favourable conditions for disease transmission, including water contamination and population displacement, ultimately escalating the risk of outbreaks and endemics. Additionally, research by Paull and colleagues [5] found that drought can amplify the spread of West Nile virus. On a larger scale, conditions aggravated by climate change, like flooding and drought, can indirectly affect disease transmission by altering ecosystems and habitats for disease vectors [6]. Understanding these climatic influences is essential for strengthening disease prevention and control strategies.

The study of historical epidemics holds immense significance spanning numerous interdisciplinary fields. Past epidemics have shaped the course of history, influencing societal structures and human development. Analysing these events provides insights into societal responses to disease and informs modern public health strategies. Historical epidemics offer a distinct epidemiological perspective, helping refine current approaches to pandemic preparedness and response. A prior study, based on past infectious disease outbreaks, emphasised the need for equitable, inclusive global health policies – ones that address the intersections of power, economics, and public health without perpetuating colonial era biases [7]. The recurrence of infectious diseases highlights the enduring relevance of historical analysis. Genomic studies of ancient pathogens also reveal the long-term co-evolution of humans and disease agents [8]. In China, historical epidemic research has deepened our understanding of disease transmission in relation to environmental and societal factors [9]. While a previous effort used the ‘A Compendium of Chinese Meteorological Records of the Last 3000 Years’ to digitise climate data for 1644–1795, it did not include corresponding epidemic records or their consequence [10]. Therefore, we seek to fill this gap.

We aim to investigate the long-term relationship between meteorological events and epidemic severity in ancient China from 674 BC to 1911 AD. By digitising and systematically analysing 2500 years of epidemic and meteorological records, we seek to quantitatively assess how floods, droughts, and famines influenced epidemic outcomes across dynastic transitions and historical contexts. Previous studies have noted potential associations between dry climates and heightened epidemic incidence, but few have offered quantitative evaluations of how specific climatic phenomena affected the severity of outbreaks over extended historical periods. [11] Addressing this gap, we developed a novel classification and scoring system to represent epidemic intensity, meteorological extremes, and their consequences, enabling multivariable statistical modelling. This empirical approach integrates historical climatology with epidemiological analysis across an unprecedented temporal scale, generating insights with relevance to both historical understanding and modern public health preparedness. Our findings may help inform contemporary strategies for epidemic risk management in an era of intensifying climate variability.

## METHODS

### Study design and data sources

We conducted a retrospective study of ancient epidemic records in China, encompassing records from the 7th century BC to the beginning of the 20th century (from the Spring and Autumn period to the Qing Dynasty). The data was retrieved from a book titled ‘A Compendium of Chinese Meteorological Records of the Last 3000 Years’ [12]. This Compendium primarily shed light on globally relevant scientific issues such as climate change, disaster prevention, and the evolution of human environments. It consisted of a systematic compilation of meteorological records spanning over 3000 years, from the 23rd century BC to 1911 AD. These records, detailing weather conditions, climatic conditions, atmospheric physical phenomena, and other related events, were sourced from a plethora of historical documents from 75 libraries in 37 cities across China. This comprehensive repository provided a rich trove of first-hand accounts that set the stage for future in-depth studies of historical climates [12]. The editorial process of ‘A Compendium of Chinese Meteorological Records of the Last 3000 Years’ adhered to strict historical textual criticism. For events recorded in multiple sources, the compilers traced the original records, removed duplicates and erroneous entries, and prioritised the earliest or most credible source. In cases where later versions provided more detailed accounts, those were included after expert comparison. When descriptions across sources diverged, preference was given to the more reliable one, and discrepancies were annotated with comments. The principle of ‘contemporaneous authors as most credible’ was followed; thus, records compiled during the Qing or Republican period describing earlier dynasties were only selectively included. Content from dubious sources was not used unless it had been previously validated. Therefore, the data set represents a critically curated historical archive, minimising common biases found in ancient records.

### Epidemics and meteorological events

We used natural language processing to extract epidemic records from the book. Two researchers (QL and CQ) independently identified and extracted information from each record, including the year of the epidemic, the name of the epidemic, any accompanying meteorological events and their descriptions, and any mentioned consequences of the epidemic, along with the specific details provided. Subsequently, two researchers (QL and SZ) independently coded all records according to the predefined classification criteria (**Table 1**). Discrepancies were resolved through discussion or adjudication by a third senior reviewer (JL). The agreement rate between the two coders was 89%, indicating a high level of consistency.

**Table 1:** Classification of epidemics, meteorological events, and consequences

	Wording
**Epidemics**	
1	Single words and phrases such as epidemic disease, plague, and specific diseases (malaria, pestilence, cholera, etc.)
2	Based on level 1, there were additional descriptions of the severity of the epidemic, such as ‘many’, ‘very’, a severe epidemic (疫甚), frequent epidemics (多疫), and an epidemic (is spreading) (疫流行).
3	The epidemic was even more severe than a level 2 epidemic: a major epidemic/pandemic (大疫), the epidemic breaks out violently (疫大作), the epidemic is raging/rampant (疫盛行/横行).
**Meteorological events**	
0	The original text did not mention any flood during the epidemic
1	Flood, drought, famine, locust, wind
2	Floods, drought, famine, locust, wind – all with greater impact)
**Consequences**	
1	Deaths <30%: ‘two to three tenths of people died’, etc.; deaths <1000: the original text specified the number of deaths, which is <1000; the epidemic had not caused any deaths, or the impact was minimal: ‘most people were sick’, etc.
2	Deaths 30–60%: ‘three tenths of people died’, ‘four tenths of people died’, ‘five to six tenths of people died’, etc.; deaths 1000–10 000: the original text specified the number of deaths, which falls between 1000 and 10 000; the epidemic had spread widely, with slightly more deaths: ‘the deceased followed one after another’, etc.
3	Deaths >60%: ‘six to seven tenths of people died’, ‘two-thirds of people died’; etc.; deaths >10 000: the original text specified the number of deaths, which exceeds 10 000; the epidemic has spread widely and resulted in a large number of deaths: ‘there were many dead, stacked on top of each other’, etc.
**NA**	The original text did not mention the consequences caused by the epidemic

Based on the wording in the record descriptions, we categorised the epidemics into three levels: level 1 – single words and phrases; level 2 – in addition to level 1, there were further descriptions of epidemic severity; level 3 – the epidemic was even more severe than a level 2 epidemic. The classification system was constructed based on classical terminology commonly found in Chinese historical epidemic records, with reference to prior historical climatology and disaster documentation studies [13]. Keywords such as a severe epidemic/pandemic (疫甚), a major epidemic (大疫), or the epidemic is raging/rampant (疫盛行) were frequently used to indicate different perceived severities and impacts and thus served as a consistent foundation for categorisation. We also classified meteorological events into three categories: 0 – the original text did not mention any accompanying events during the epidemic; 1 – flood, drought, famine, locust, and wind; 2 – floods, droughts, famines, locusts, and winds, all having a greater impact.

Interpreting the consequences of an epidemic was a complex process. We summarised them into three categories, including: the proportion of fatalities, the specific number of fatalities, and textual descriptions. Each category was also divided into three levels, and the consequences of the same level were grouped (**Table 1**). Epidemic records that did not describe the consequences were marked as NA (Table S1 in the **Online Supplementary Document**).

### Statistical analysis

We described the research results on scales of 100 years, ten years, and per year. For every hundred years, we have counted the occurrences of epidemics and their associated meteorological events. We performed similar accumulations for every ten years and calculated the proportion of all epidemics within these periods that were accompanied by a meteorological event. Based on these percentages, we calculated the estimated decennial percentage change, a commonly used metric to quantify trend proportions over a specific interval [14,15]. We fitted a regression line to the natural logarithm of the proportions (*y = α* + *βx* + ε, where y = ln(rate) and x = time). We calculated the estimated decennial percentage change as (e^β^ − 1) × 100% with 95% confidence intervals (CIs) obtained from the linear regression model. As a sensitivity check, we also visualised the trend using non-parametric locally weighted scatterplot smoothing.

To reduce potential subjectivity in interpreting individual historical records, we aggregated all epidemic-related entries within the same calendar year into a single composite record. Rather than simple event counting, we summed the digitised severity levels to generate an annual epidemic index. For example, if there were one level 1 and one level 2 epidemic in a given year, the epidemic index for that year would be three; the same applies to the meteorological events and consequence indices. Subsequently, we categorised all years with epidemic records into three categories: mild (0), moderate (1–10), and severe (>10), based on the consequence index. Likewise, we divided the overall yearly epidemic severity into three levels: mild (1–3), moderate (4–15), and severe (>15), based on the epidemic index. The meteorological events were not subject to further processing. Finally, we employed a multivariable logistic regression model to explore the effect of meteorological events occurring within a given year on the consequences of that year’s epidemic events. The formula was as follows: *logit*(*P*(*Y* = 1|*X*)) =  *β_0_* + *β_1_epiSeverity* + *β_2_flood* + *β_3_drought* + *β_4_famine* + *β_5_locusts* + *β_6_wind* + *β_7_dynasty*

where *Y* denotes the epidemic consequence category, with separate models constructed for two binary comparisons: mild *vs.* moderate and moderate *vs.* severe. The predictor epiSeverity represents the categorised epidemic severity index. Flood, drought, famine, locusts, and wind are meteorological event frequencies, and dynasty is a categorical variable indicating historical period (Before Ming, Ming, Qing).

We performed all analyses using *R*, version 4.3.2 (R Core Team, Vienna, Austria).

## RESULTS

### Overall analysis of epidemic records and meteorological impact

We extracted 5338 records containing epidemic information from ‘A Compendium of Chinese Meteorological Records’, among which 247 records were from before the Ming dynasty, 1898 from the Ming dynasty, and 3193 from the Qing dynasty. When viewed in hundred-year increments (given the scarcity of records before BC, all records from this era were aggregated; the records are only maintained until 1911, rendering the final category as 1901–1911), most epidemic incidents were catalogued after the year 1400. The 19th century logged the most incidents, with 757 level 1, 52 level 2, and 939 level 3 records (**Figure 1**, Panel A). The meteorological events most frequently recorded in conjunction with these epidemics were famine (n = 611), drought (n = 564), and floods (n = 245). The 17th century witnessed the greatest number of recorded famines (n = 173), followed closely by the 16th century (n = 168) (**Figure 1**, Panel B). The most drought events were recorded in the 16th century (n = 180), followed by the 17th century (n = 157). The largest number of floods was reported in the 19th century (n = 86), followed by the 16th century (n = 46). It is important to note that the post-1400 increase in epidemic records may partially reflect improved documentation practices rather than an actual rise in epidemic frequency.

Examining the proportion of epidemics accompanied by meteorological events on a decade basis, it was observed that although the number of recorded epidemics increased over time, the proportion of these epidemics associated with meteorological events decreased at a rate of 0.24% (95% CI = 0.17–0.31) per decade from 1451 to 1911, descending from 65.38% to 23.08% (**Figure 2**, Panel C). Before the Ming Dynasty, there were periods when 100% of epidemics were linked to meteorological events (**Figure 2**, Panel A), likely due to the scarcity of records (most recorded fewer than three records). During the Ming and Qing dynasties, the highest recorded proportion of epidemics associated with meteorological events was between 1451 and 1460 at 65.38% (n = 17/N = 26 cases), followed by 48.15% (n = 26/N = 54 cases) in 1481–1490, and 44.35% (n = 157/N = 354 cases) in 1581–1590. (**Figure 2**, Panel B) For a sensitivity analysis, we applied locally weighted scatterplot smoothing to visualise the trend in the decadal proportion of epidemics associated with meteorological phenomena (Figure S1 in the **Online Supplementary Document**). The smoothed curve showed a marked decline beginning around the year 1500, consistent with the estimated decennial percentage change results. This supports the robustness of the observed downward trend, particularly during the Ming and Qing periods when historical records became more complete and standardised.

### Impact of meteorological events on the epidemic consequences

There were 212 level 1, 652 level 2, and 912 level 3 records (**Table 2**). Of the records with level 1 consequences, the majority (n = 140; 66.04%) were constituted by level 1 epidemics. Records with level 2 (n = 334; 51.23%) and level 3 (n = 443; 48.75%) consequences, however, were dominated by level 3 epidemics. In all three levels of consequences, there were few records of floods, with ten in level 1, 11 in level 2, and 48 in level 3, of which six (2.83%) in level 1, six (0.92%) in level 2, and 21 (2.30%) in level 3 had major impacts. For drought records, there were 13 in level 1, 29 in level 2, and 151 in level 3 consequences, of which five (2.36%) in level 1, 11 (1.69%) in level 2, and 85 (9.32%) in level 3 had significant impacts. Lastly, for famine records, there were 15 in level 1, 41 in level 2, and 189 in level 3 consequences, with eight (3.77%) in level 1, 20 (1.53%) in level 2, and 101 (11.07%) in level 3 having significant impacts. In all records of any level of consequence, occurrences of locust plague and wind disasters were relatively few.

**Table 2:** Distribution of epidemic consequences and related meteorological events*

	Level 1 (n = 212)	Level 2 (n = 652)	Level 3 (n = 912)
**Epidemics**			
1	140 (66.04)	291 (44.63)	437 (47.92)
2	8 (3.77)	27 (4.14)	32 (3.51)
3	64 (30.19)	334 (51.23)	443 (48.75)
**Flood**			
1	4 (1.89)	5 (0.77)	27 (2.96)
2	6 (2.83)	6 (0.92)	21 (2.30)
**Drought**			
1	8 (3.77)	18 (2.76)	66 (7.24)
2	5 (2.36)	11 (1.69)	85 (9.32)
**Famine**			
1	7 (3.30)	21 (3.22)	88 (9.65)
2	8 (3.77)	20 (1.53)	101 (11.07)
**Locust**			
1	1 (0.47)	7 (1.07)	26 (2.85)
2	0	0	0
**Wind**			
1	0	1 (0.15)	0
2	0	0	4 (0.44)

*Values are presented as numbers (percentages).

In the 275 years categorised as having mild consequences (*i.e.* consequence index = 0), the frequency of meteorological events was predominantly zero. Among these, the highest occurrences of floods were in 1492 (n = 4), droughts in 1501 (n = 5), and famines in 1452 (n = 5). In the 278 years characterised as moderate consequences (*i.e.* 0<consequence index <10), despite most of the years having a zero frequency for all types of meteorological events, the proportion and frequency of occurrence of meteorological events still showed an increase. Excluding years with a frequency of zero, the years with a flood frequency of two were the most common, occurring 37 times. The highest frequency remained at four times but occurred in eight different years. The drought frequency of one was most common, occurring in 46 years, with the highest frequency being 12 times in 1482. Famine had a frequency of one most often, appearing in 43 years, with the highest frequency being nine times in 1508. The 103 years classified as severe consequences (*i.e.* consequence index >10) saw a significant increase in the frequency of meteorological events: only 51 years (49.51%) had no floods, with the highest flood frequency being 25 times in 1821, 76 years (73.79%) experienced droughts, with the most frequent being 62 times in 1641, and 86 years (83.50%) experienced famines, with the highest frequency at 65 times in 1641. The most common levels of epidemic severity were level 1 in mild consequences (n = 190/N = 275; 69.09%), level 2 in moderate consequences (n = 135/N = 278; 48.56%), and level 3 in severe consequences (n = 95/N = 103; 92.23%) (**Figure 3**, Panels A–C).

In the multinomial logistic regression model, we found that the severity of epidemics in a given year, along with the occurrence of floods, droughts, and famines, was a predictor of the severity of epidemic consequences that year (**Table 3**). After controlling for other factors, for each increase in the severity level of the yearly epidemic, the likelihood of the epidemic resulting in moderate consequences was 5.01 times (95% CI = 3.28–7.66) greater than it causing mild consequences. For each additional occurrence of a flood, the likelihood of the epidemic leading to a moderate consequence had increased by 42% (adjusted odds ratio (aOR) = 1.42; 95% CI = 1.08–1.87) compared to causing a mild consequence. Every additional famine increased the likelihood by 40% (aOR = 1.40; 95% CI = 1.11–1.76). The influences of drought, locust, and wind were not statistically significant. When comparing the risk of an epidemic resulting in severe consequences (as opposed to moderate consequences) in a given year, each increase in the severity level of epidemics had increased this risk by 14.53 times (95% CI = 5.76–36.64). Each additional occurrence of a flood (aOR = 1.46; 95% CI = 1.12–1.91), drought (aOR = 1.23; 95% CI = 1.07–1.41), and famine (aOR = 1.55; 95% CI = 1.31–1.82) increased this risk. The impacts of a locust and wind on this risk were not statistically significant.

**Table 3:** Multinomial logistic regression results for the severity of epidemic consequences

	Moderate (ref: mild)		Severe (ref: moderate)	
	**aOR (95% CI)**	***P*-value**	**aOR (95% CI)**	***P*-value**
**Severity of epidemics**	5.01 (3.28–7.66)	<0.001	14.53 (5.76–36.64)	<0.001
**Flood**	1.42 (1.08–1.87)	0.013	1.46 (1.12–1.91)	0.006
**Drought**	1.21 (0.97–1.52)	0.087	1.23 (1.07–1.41)	0.003
**Famine**	1.40 (1.11–1.76)	0.004	1.55 (1.31–1.82)	<0.001
**Locusts**	1.17 (0.46–2.92)	0.734	1.15 (0.68–1.96)	0.607
**Wind**	1.41 (0.65–3.05)	0.389	0.78 (0.32–1.87)	0.578

aOR – adjusted odds ratio, CI – confidence interval, ref – reference

## DISCUSSION

To our knowledge, this is the first study to systematically quantify the long-term relationship between epidemics and meteorological events, such as floods, droughts, and famines, using archival records spanning over two millennia. Our findings show that although the number of recorded epidemics increased over time, the proportion associated with meteorological events declined. Epidemic severity and the occurrence of floods, droughts, and famines in a given year were significant predictors of epidemic consequences. These results support the hypothesis that meteorological stressors exacerbate epidemic severity. Our results enhance our understanding of how environmental shocks have historically influenced disease dynamics and offer insight into how such relationships might inform present-day epidemic risk assessments. As climate change continues to intensify the frequency and severity of extreme weather events, recognising their potential role in amplifying disease impacts is increasingly important for public health preparedness.

Our results showed that while the number of recorded epidemics over the course of ancient Chinese history increased, there was a declining trend in the proportion of these epidemics associated with meteorological events. On one hand, the increase in recorded epidemics could be attributed to advancements in documentation and a heightened understanding of diseases over time, which could have resulted in increased reporting [16]. On the other hand, the diminishing trend of meteorologically linked epidemics might reflect multiple underlying factors. One possibility is an evolving landscape of disease aetiology, such as increased societal resilience to climate-based disease triggers [17], or the emergence of non-climate-related disease factors (*e.g.* behavioural or socioeconomic changes). Alternatively, this trend could also be influenced by shifts in historical narrative emphasis, selective documentation practices, or a decline in the inclusion of meteorological detail in epidemic records over time. Given these possibilities, we interpret this pattern with caution. The dissection of these contrasting trends enables a comprehensive understanding of disease-causing factors and their transformation over the centuries. While causality cannot be established, these insights may still help inform modern public health strategies by contextualising how the relationship between environmental stressors and epidemic outcomes may evolve.

We found a significant association between annual flood occurrence and epidemic consequences. Each additional flood event increased the odds of a moderate consequence (*vs.* mild) by 42%, and a severe consequence (*vs.* moderate) by 46%. These findings align with previous studies. For instance, floods have been shown to intensify dysentery epidemics [18], and adverse health impacts have been linked to flood events, though seasonal effects were not separately analysed [19]. This highlights the crucial role of flood management in reducing the severity of epidemics. Effective interventions require international collaboration, particularly since prior research has shown that low-income countries bear a disproportionate share of the health burdens from flood-related disasters [20]. Providing economic support and helping these countries develop disaster risk analysis systems could strengthen preparedness, guide evidence-based policy, and identify drivers of vulnerability across regions [21,22].

Drought frequency was also significantly associated with epidemic severity. Each additional drought event in a given year increased the odds of severe consequences (*vs.* moderate) by 23%, although no significant difference was found between mild and moderate outcomes. This aligns with prior studies suggesting that the socioeconomic context of droughts influences community vulnerability and epidemic intensity [23]. For example, Stone’s examination of fourteenth-century England emphasised the longstanding link between drought and heightened epidemic severity [24]. These results underscore the need to explore drought-epidemic interactions through mixed-method approaches, incorporating both climatic and social dimensions. Understanding the mechanisms behind these associations is crucial for enhancing risk prediction and informing public health response, particularly in regions prone to climate extremes. Famines also emerged as a major factor associated with increased epidemic severity, consistent with both historical literature and our findings. In ancient Chinese records, famines – alongside droughts – were the most frequently co-reported meteorological events in epidemic years [25]. Famine contributes to epidemics through multiple pathways, including population displacement, malnutrition-induced immunosuppression, and increased social instability [26–28]. These findings emphasise the need to integrate climate-related stressors such as famine and drought into health preparedness strategies. Public health policies should aim to strengthen system resilience, protect vulnerable populations, and coordinate climate adaptation with epidemic prevention. Further research is needed to clarify causal pathways and support targeted intervention in climate-vulnerable regions.

Notably, our findings also revealed historical periods where severe meteorological events occurred without leading to high-severity epidemics. For example, several years categorised with high frequencies of flood or drought events were still associated with only mild or moderate epidemic consequences. This suggests that the relationship between climate stressors and epidemic severity is not deterministic. Numerous studies affirm that epidemic outcomes amid environmental stressors are highly mediated by contextual factors, including local governance capacity, effectiveness of disaster relief and infrastructure, patterns of population displacement, and levels of social cohesion or community resilience [29–31]. These mediating factors shape a population’s vulnerability and adaptive response capacity. These inconsistencies highlight the nonlinear and socially modulated nature of the climate-health nexus, underscoring the importance of contextual resilience in determining epidemic severity under environmental threats.

Our findings confirm broader patterns observed in historical epidemiological research. Globally, studies have documented the role of environmental shocks – such as droughts, floods, and famines – in amplifying epidemic risks in pre-modern societies. For instance, research from Europe has linked famine-induced malnutrition to increased plague mortality during the Black Death and subsequent outbreaks [32]. Studies in colonial India and sub-Saharan Africa have similarly shown how climatic extremes weakened population resilience and heightened vulnerability to infectious diseases [33,34]. These patterns suggest that the climate-epidemic linkage we observe in ancient China may reflect a more universal phenomenon shaped by environmental exposure and social response capacity. Integrating perspectives from historical epidemiology and climate-history scholarship thus reinforces the relevance of our findings beyond the Chinese context and offers a valuable framework for understanding climate-sensitive health risks across cultures and time periods.

Our study had some limitations. First, our findings are based on historical records, which may have been subject to potential subjective judgment and limited understanding at the time of documentation. Although the Compendium was compiled with rigorous textual scrutiny, potential biases – such as underreporting during certain dynasties or inconsistent regional documentation – may still influence the results. Triangulation with local chronicles, imperial archives, or archaeological data would further enhance the robustness of the data set. Second, most epidemic records did not specify causative pathogens or disease types, limiting our ability to analyse climate sensitivity for specific diseases. Third, the increase in epidemic reports after 1400 may reflect improved documentation rather than actual epidemiological trends. While normalisation by population or literacy rates could mitigate this bias, the lack of reliable demographic data across 2500 years presents substantial challenges. Future studies may consider using dynastic-level population reconstructions or archival survival rates as proxies. Fourth, aggregating multiple epidemic events into a single annual composite index may obscure heterogeneity across outbreaks. Although this approach helps reduce subjectivity in individual record interpretation, it may conflate distinct events. Future work could consider stratified or event-level analyses if spatial or etiological detail is available. Fifth, key socio-economic confounders – such as population density, trade activity, governance shifts, or conflict exposure –were not included due to limited historical data availability. These factors may independently influence both epidemic and meteorological patterns; therefore, the future integration of external historical data sets is encouraged. Sixth, we assumed linear trends in our decennial percentage change analysis, which may oversimplify complex, non-stationary dynamics in long-term historical processes. To partially address this, we conducted a locally weighted scatterplot smoothing-based sensitivity analysis (Figure S1 in the **Online Supplementary Document**), which confirmed the robustness of the overall declining trend. Seventh, we did not explicitly model temporal autocorrelation in the regression analysis. Epidemics may have clustered in certain periods due to unobserved shocks (*e.g.* wars, migrations), which could bias the estimated effects. Given the irregular structure of the data, time-series or mixed-effects models could be explored in future research. Finally, classification of epidemic and meteorological severity relied on qualitative textual descriptions. Despite implementing structured coding and independent review, interpretation bias may remain.

## CONCLUSIONS

Our research on the long-term historical interactions between meteorological events and epidemics offers valuable insights into modern epidemic prevention and control. While our findings showed that floods, droughts and famines were significantly associated with greater epidemic severity, this relationship was not universally consistent across all historical periods. Certain meteorological extremes did not correspond with heightened epidemic outcomes, suggesting that the impact of climate shocks was likely moderated by contextual factors such as governance structures, population responses, and disaster preparedness. These historical inconsistencies highlight the importance of resilience mechanisms in shaping epidemic outcomes, reminding us that climate is only one part of a broader vulnerability landscape. As the global climate continues to shift, understanding and predicting how these events influence disease propagation becomes pivotal for public health. Regions particularly vulnerable to climate change may require tailored epidemic prevention and control strategies that account for potential climatic extremes. Learning from history can thus strengthen our capacity to anticipate and manage epidemic risks associated with meteorological events, contributing to more targeted, adaptive, and effective health strategies.

## Additional Material


Online Supplementary Document

**Figure 1.** Distribution and frequency of epidemics and accompanying meteorological events. **Panel A.** Epidemics. **Panel B.** Meteorological events.

**Figure 2.** Decadal trends in the proportion of epidemics accompanied by meteorological events. **Panel A.** Before Ming. **Panel B.** Ming & Qing. **Panel C.** Percentage of meteorological phenomena.

**Figure 3.** Meteorological event frequencies and levels of epidemic severity under different consequence categories. **Panel A.** Mild consequence. **Panel B.** Moderate consequence. **Panel C.** Severe consequence.
