# Supplementary material for: Trends of infectious diseases, epidemic patterns, and the association with meteorological events: 2500 years of evidence from an observational study
Source: J Glob Health. 2025 Sep 5;15:04254. doi: 10.7189/jogh.15.04254 (PMC12412270; doi:10.7189/jogh.15.04254)

Supplement to: Qiao L, Chenyuan Q, Shimo Z, Jue L. Trends of infectious diseases, epidemic patterns, and the association with meteorological events: 2500 years of evidence from an observational study. J Glob Health. 2025;15:04254.

Table S1. Examples of record digitization and index assignment based on historical texts

Original Historical Text	Epidemic Level	Drought	Famine	Flood	Locust	Wind	Consequence Level	Notes
十月，租长陵田。大旱。衡山国、河东、云中郡民疫	1	2	0	0	0	0	0	“疫” indicates a general epidemic reference → Epidemic Level 1; “大旱” denotes severe drought → Drought Level 2; no consequence described → Consequence Level 0.
泰宁县夏饥。秋。疫死以千计。	1	0	1	0	0	0	2	“疫” implies a basic epidemic reference → Epidemic Level 1; “饥” indicates moderate famine → Famine Level 1; “死以千计” refers to deaths in the thousands → Consequence Level 2.
时疫大行，亡者枕藉，骨肉不相顾。	2	0	0	0	0	0	3	“时疫大行” suggests widespread epidemic → Epidemic Level 2; “亡者枕藉” reflects mass death → Consequence Level 3; no meteorological event mentioned → all weather indices = 0.

Figure S1. Trend of meteorologically-associated epidemics (10-year aggregation)

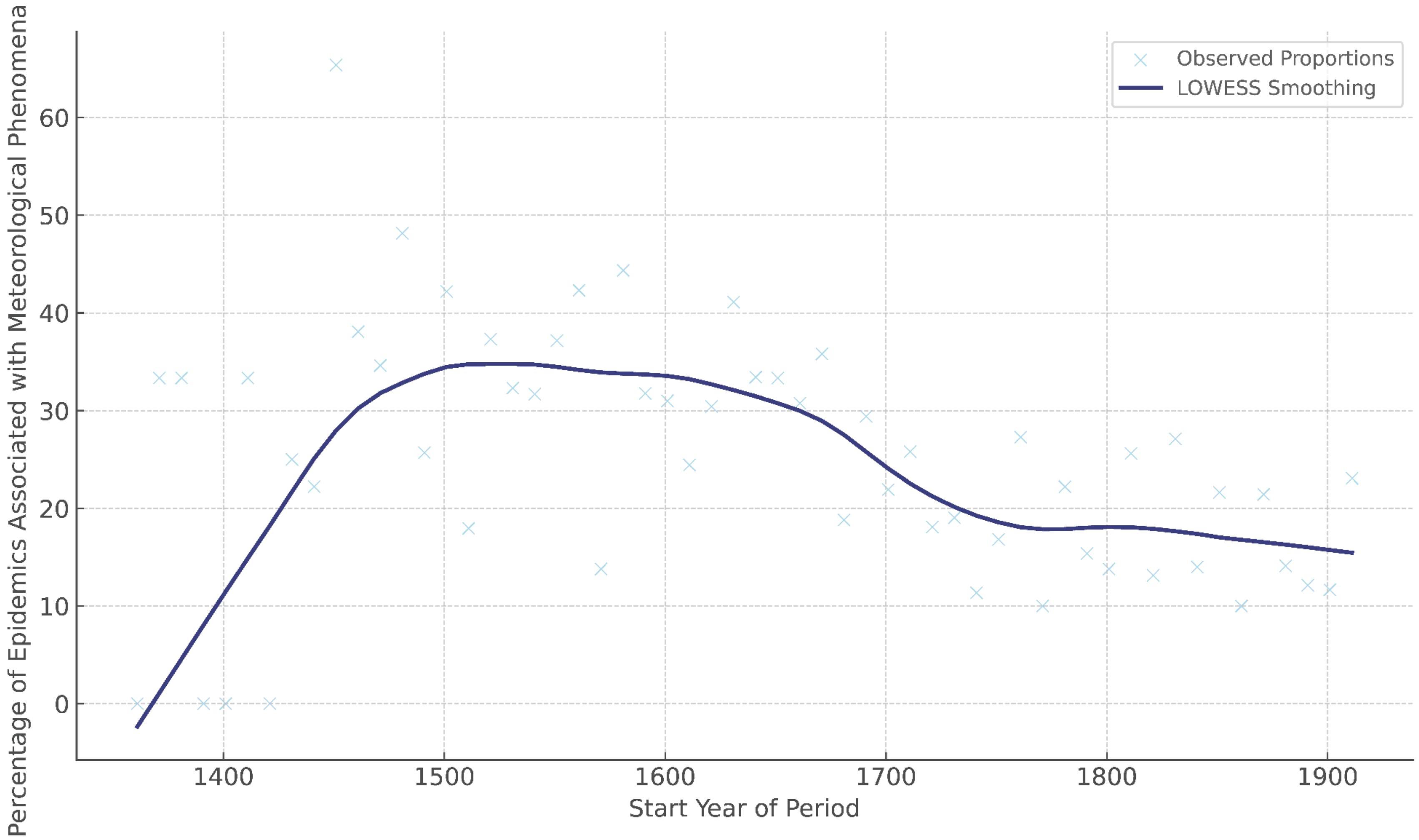

Supplement: Online Supplementary Document [file jogh-15-04254-s001.pdf]
